# George Baillie on peptide array, a technique that transformed research on phosphodiesterases

**DOI:** 10.4155/fso.15.25

**Published:** 2015-11-01

**Authors:** George S Baillie

**Affiliations:** 1 Institute of Cardiovascular and Medical Sciences, CMVLS, Glasgow University, Glasgow, UK

## Abstract

**George Baillie speaks to Francesca Lake (Managing Editor, *Future Science OA*):** George Baillie is a Professor and PI within the Institute of Cardiovascular and Medical Sciences at the University of Glasgow (Glasgow, UK). His research over the last 15 years has examined many aspects of the cAMP signaling pathway in disease and he has published over 140 papers on the subject. His major discovery was that phosphodiesterases are ‘compartmentalized’, and it is their location within cells that direct their function. The Baillie/Houslay laboratory was the first to discover a specific function for a single isoform of PDE4 (namely PDE4D5 with β-arrestin desensitizes the β2-adrenergic receptor). His laboratory has since gone on to ascribe functions to several other PDE4 isoforms. He is a founder and director of Sannox Therapeutics, a spin-out venture within University of Glasgow. He is also a member of the Editorial Board of *Future Science OA and Co-Editor of Cellular Signalling*.

## Q Could you tell our readers a little about your background & current position at the University of Glasgow?

I am a professor of molecular pharmacology with a medium-sized research group. I started at Glasgow University as a technician and worked my way up to post-doc, and then through to lecturer, reader and professor. I have been here about 23 years, and just worked my way up from the beginning. I do everything – teaching, admin, running my own research group – a bit of everything.

## Q Your first degree was in agricultural botany; what made you shift focus & complete your PhD in pharmacology?

I did my PhD with Pfizer down in Sandwich (UK), which is when I got into drug discovery. I was working on agricultural botany – I was interested in potatoes, and how to stop them getting contaminated with fungal disease when they are stored. I wanted to understand how they get the fungal disease. I am from Ayrshire in Scotland, one of the biggest potato-growing regions, and I was heavily into that because of my local area, but it did not turn out that way. I actually tried to get a PhD, which I wrote myself, on the storage of potatoes, but I did not get the funding. I went to work for Pfizer on an antifungal compound, but actually for humans. So I went from fungal disease into antifungals, into drug discovery. That is basically how I got here.

## Q What is the focus of your current research?

We work predominantly on phosphodiesterases. Most of my group works on cAMP-signaling pathways and that means we work on many different diseases. We have got a funded project on chronic obstructive pulmonary disease (COPD) from the Medical Research Council, a schizophrenia-funded project, which is soon coming to its end, funded by Scottish Enterprise, and a couple of people working on prostate cancer, as well. These are all areas in which cAMP is involved. So we are pathway specific, rather than disease specific.

## Q What have you found with the schizophrenia project?

We have found a novel way to restore the levels of an important signal scaffold protein called DISC1 that is involved in synaptic processes and brain development. This protein is downregulated in some forms of schizophrenia and bipolar disease. We can now interfere with a regulatory pathway that controls protein levels of DISC1 and restore the deficit in patient derived cells.

## Q Your laboratory is a ‘world leader’ in using peptide array to map protein–protein interactions. Can you tell us about this?

We decided to make a novel PDE4 inhibitor, that didn't target the active site – it targeted the area in the cell where the protein was anchored. So we started working on protein–protein interactions of phosphodiesterases, and how they are compartmentalized in cells by their anchoring domain. There are many isoforms and the only unique part is the anchoring domain. They essentially do the same thing, but in different locations. To do that, we asked someone who knew about peptide array to map protein–protein interactions to help, and it was so successful we stopped doing things with yeast two-hybrid and with other molecular techniques. To map a protein–protein interaction over 25 amino acids, it takes a week using peptide array but over 6 months using yeast two-hybrid. We usually get the same results.

Basically, it is a robot that makes peptide on a cellulose matrix. The peptides are covalently linked to the cellulose fibers; it anchors them so you can make peptide libraries of any protein sequence on surfaces, and then you can put other proteins over the top, and see what peptides they bind to. We have got two different types of robots, and we are the only people in Scotland with the capability.

## Q What do you think are the biggest challenges in your research area at present?

Funding is very difficult at the moment. Our research is mostly translational, and we find it difficult getting money for translational research. That is starting to change. The British Heart Foundation has just announced its translational grant scheme, and the Medical Research Council has their own and there are various other ones. We do drug discovery, so it is quite a challenge getting the money in for that.

## Q Why is translational research lagging behind when it comes to funding?

Traditionally, the UK has funded basic research, and now the impact agenda is changing that – people need an output from their research; they need to show that they have made a discovery that is going to change something. With our translational research, we are better aligned to that, so things are starting to change, although it has been a slow process.

## Q How do you see this changing over the next 10 years or so?

I think more and more of the funding bodies are realizing that there has to be some sort of impact from research, and it is got to be measurable. One of the things we can do is actually utilize our basic research, convert that into drug discovery and, hopefully, one day we might have a compound that is derived from our research. I do have a spin-off company that is trying to take some of these things forward that we have done. That is Sannox Therapeutics, founded last year with the help of Glasgow University. Sannox intends to take some of the compounds and peptides that we have discovered that disrupt protein–protein interactions and commercialise them. Basically, we need more information about how our compounds and peptides work to make them attractive to pharmaceutical companies. It is very early days yet.

## Q What would you say has been your greatest achievement in your career to date?

In science terms, I was involved in the discovery that a protein called β-arrestin binds to phosphodiesterases and takes them to receptors to get rid of cAMP that has been produced as a result of this receptor activation – it was a receptor desensitization step. It was a paper with Bob Lefkowitz, who won the Nobel Prize recently for his work on receptors. So we actually published with him on that discovery, which I think is my biggest achievement!

## Q You have various extra roles, including teaching & being a ‘Senate Assessor on Court’ at Glasgow University; can you tell us about these?

If you are a professor, you are on the Senate. Basically, all of the major decisions that happen at the University have to be put past the Senate for its approval. As there are so many professors, there are seven assessors selected by the Senate, who sit in on high-level management meetings to represent the Senate's interest. Basically, we sit on University Court, major panels of recruitment, reviews for periodical change of subject areas, the financial and HR committees and we represent Senate's interests in all these major committees. We read all the papers, get our heads round why all decisions have been made and challenge them if we do not think they are right. I was recently involved in the periodical review for Urban Studies – you are not allowed to be involved in reviews within your subject area – so I get to see how social science institutes work, which is completely different to ours and very interesting.

I also do lots of teaching, at all levels and a number of different courses, from basic protein structure to RNA and DNA in the first year, right through to complex phosphodiesterase function in the final year. I do laboratories, lectures, tutorials – I have approximately 65 contact hours. I am also a course coordinator for the cell signaling option in the fourth year. I am in very early!

## Q You have recently been appointed to the *Future Science OA* editorial board; what are your thoughts on open access?

I think it is debatable. I think we will end up open access in the end. There is no point making a discovery if people cannot find out about it. I think journals like *Future Science OA* are the way to go, and I think because they are easier to find, they will get more hits, become more popular and everyone else will have to follow suit.

## Q Finally, if you had unlimited resources, what would you do & why?

Peptide array can only really be used for protein–protein interactions that happen over a very short, contiguous sequence, so if the proteins bind in regions formed by 3D structures from different parts of the protein then we cannot find it. Basically, there are certain types of protein–protein interaction ideal for peptide array. One of them is E3 ligase/substrate interactions in the ubiquitin cascade. They always bind onto short docking sequences before they trigger ubiquitination of the protein. So, what I would do, is take all of the orphan E3 ligases and set up yeast two-hybrid screens to find the substrates, and then I would find the binding sites using peptide array, and I would make competing peptides and use those peptides to undergo drug discovery to find E3 ligase inhibitors for all the different classes of E3 ligase. There are hundreds and hundreds of them, so it would take a lot of time and money!

